# Retraction Note to: Chronic NMDA administration to rats increases brain pro-apoptotic factors while decreasing anti-Apoptotic factors and causes cell death

**DOI:** 10.1186/s12868-017-0359-y

**Published:** 2017-05-09

**Authors:** Hyung-Wook Kim, Yunyoung C Chang, Mei Chen, Stanley I Rapoport, Jagadeesh S Rao

**Affiliations:** Brain Physiology and Metabolism Section, National Institute on Aging, National Institutes of Health, Bethesda, MD 20892 USA

## Retraction Note to: BMC Neuroscience 2009, 10:123 DOI 10.1186/1471-2202-10-123

This article [1] has been retracted by the editor because author Stanley I Rapoport alerted the editor, and the National Institutes of Health subsequently confirmed, that the data represented by Figures 1B and 2B were falsified. Stanley I Rapoport and Hyung-Wook Kim support this retraction. The other authors have not responded to our correspondence with them about the retraction of their article.
